# Association of Infants Exposed to Prenatal Zika Virus Infection With Their Clinical, Neurologic, and Developmental Status Evaluated via the General Movement Assessment Tool

**DOI:** 10.1001/jamanetworkopen.2018.7235

**Published:** 2019-01-18

**Authors:** Christa Einspieler, Fabiana Utsch, Patricia Brasil, Carolina Y. Panvequio Aizawa, Colleen Peyton, Renata Hydee Hasue, Fernanda Françoso Genovesi, Luana Damasceno, Maria Elisabeth Moreira, Kristina Adachi, Peter B. Marschik, Karin Nielsen-Saines

**Affiliations:** 1 Interdisciplinary Developmental Neuroscience–iDN, Medical University of Graz, Graz, Austria; 2 Rede SARAH de Hospitais de Reabilitação, Reabilitação Infantil, Belo Horizonte, Brazil; 3 Laboratorio de Doenças Febris Agudas, Instituto Nacional de Infectologia Evandro Chagas (INI), Fundação Oswaldo Cruz (Fiocruz), Rio de Janeiro, Brazil; 4 Department of Physical Therapy, Communication Sciences & Disorders, and Occupational Therapy, Faculty of Medicine, University of São Paulo, São Paulo, Brazil; 5 Department of Physical Therapy and Human Movement Science, Northwestern University, Chicago, Illinois; 6 Department of Pediatrics, University of Chicago, Chicago, Illinois; 7 Department of Pediatrics, Instituto Fernandes Figueira, Fiocruz, Rio de Janeiro, Brazil; 8 Division of Pediatric Infectious Diseases, Department of Pediatrics, David Geffen School of Medicine, University of California, Los Angeles; 9 Interdisciplinary Developmental Neuroscience–iDN, Department of Child and Adolescent Psychiatry and Psychotherapy, University Medical Center Göttingen, Göttingen, Germany; 10 Center of Neurodevelopmental Disorders (KIND), Department of Women’s and Children’s Health, Karolinska Institutet, Stockholm, Sweden

## Abstract

**Importance:**

There is an urgent need to assess neurodevelopment in Zika virus (ZIKV)–exposed infants.

**Objectives:**

To perform general movement assessment (GMA) at 9 to 20 weeks’ postterm age and to evaluate whether the findings are associated with neurodevelopmental outcomes at age 12 months in infants prenatally exposed to acute maternal illness with rash in Brazil during the ZIKV outbreak and in age-matched controls.

**Design, Setting, and Participants:**

In this cohort study, infants prenatally exposed to acute maternal illness with rash were recruited at medical institutions in Rio de Janeiro and Belo Horizonte, Brazil, from February 1, 2016, to April 30, 2017, while infants without any exposure to maternal illness originated from the Graz University Audiovisual Research Database for the Interdisciplinary Analysis of Neurodevelopment. Participants were 444 infants, including 76 infants without congenital microcephaly, 35 infants with microcephaly, and 333 neurotypical children matched for sex, gestational age at birth, and age at GMA.

**Main Outcomes and Measures:**

General movement assessment performed at 9 to 20 weeks’ postterm age, with negative predictive value, positive predictive value, sensitivity, and specificity generated, as well as clinical, neurologic, and developmental status (Bayley Scales of Infant and Toddler Development, Third Edition [Bayley-III] scores) at age 12 months. Motor Optimality Scores were generated based on the overall quality of the motor repertoire. Adverse outcomes were defined as a Bayley-III score less than 2 SD in at least 1 domain, a score less than 1 SD in at least 2 domains, and/or atypical neurologic findings.

**Results:**

A total of 444 infants were enrolled, including 111 children prenatally exposed to a maternal illness with rash and 333 children without any prenatal exposure to maternal illness (57.7% male and mean [SD] age, 14 [2] weeks for both groups); 82.1% (46 of 56) of ZIKV-exposed infants without congenital microcephaly were healthy at age 12 months. Forty-four of 46 infants were correctly identified by GMA at 3 months, with a negative predictive value of 94% (95% CI, 85%-97%). Seven of 10 ZIKV-exposed children without microcephaly with adverse neurodevelopmental outcomes were identified by GMA. The GMA positive predictive value was 78% (95% CI, 46%-94%), sensitivity was 70% (95% CI, 35%-93%), specificity was 96% (95% CI, 85%-99%), and accuracy was 91% (95% CI, 80%-97%). Children with microcephaly had bilateral spastic cerebral palsy; none had normal movements. The Motor Optimality Score differentiated outcomes: the median Motor Optimality Score was 23 (interquartile range [IQR], 21-26) in children with normal development, 12 (IQR, 8-19) in children with adverse outcomes, and 5 (IQR, 5-6) in children with microcephaly, a significant difference (*P* = .001).

**Conclusions and Relevance:**

This study suggests that although a large proportion of ZIKV-exposed infants without microcephaly develop normally, many do not. The GMA should be incorporated into routine infant assessments to enable early entry into targeted treatment programs.

## Key Points

**Question:**

Is there an association between general movement assessment results and neurodevelopment in infants with vertical Zika virus exposure?

**Findings:**

In this cohort study of 444 children, including 111 prenatally exposed to acute maternal illness with rash during the Zika epidemic, general movement assessment was associated with neurodevelopment at age 12 months (94% negative predictive value, 78% positive predictive value, 70% sensitivity, 96% specificity, and 91% accuracy). The Motor Optimality Score was 23 in children with normal development, 12 in children with adverse outcomes, and 5 in children with microcephaly, a significant difference.

**Meaning:**

General movement assessment is a helpful tool in the evaluation of neurodevelopment in Zika virus–exposed children.

## Teaser

This cohort study of infants prenatally exposed to acute maternal illness with rash in Brazil during the Zika virus outbreak examines results of a general movement assessment performed at 9 to 20 weeks’ postterm age and evaluates whether the findings are associated with neurodevelopmental outcomes at age 12 months compared with age-matched controls.

## Introduction

Symptomatic Zika virus (ZIKV) infection of pregnant women is characterized by an acute illness with fatigue and malaise, arthralgia, conjunctivitis, and most characteristically a pruritic rash.^1,2,3,4^ Similar symptoms also occur in other mosquito-borne flavivirus infections, such as dengue or chikungunya. Differential diagnosis requires real-time reverse transcriptase–polymerase chain reaction (RT-PCR) assays performed on blood, urine, or both during the acute period of illness.^5^ Serologic testing often poses problems in areas where Zika and dengue viruses cocirculate due to serologic cross-reactivity.

After the 2015-2016 ZIKV epidemic in Brazil, the Americas, and beyond, as well as recognition of the associated neurologic findings in infants with vertical exposure to ZIKV,^3,6,7^ we were confronted with the urgent need to answer multiple questions. What proportion of infants have normal neurologic outcomes after prenatal exposure to maternal Zika infection? At what age can families be reassured that their child will develop normally? Is it possible to anticipate neurologic sequelae? When should early interventions start?

The application of a reliable and valid diagnostic tool, such as the general movement assessment (GMA), to functionally assess the integrity of the developing nervous system may yield particular insights.^8,9^ The GMA, a gestalt-based observational method to classify early motor functions in the first months of life, has shown merits for the prediction of both normal neurodevelopment and adverse outcomes.^8,10,11^

In this study, we aimed to assess the integrity of the developing nervous system by analyzing the neuromotor repertoire of infants prenatally exposed to ZIKV. In a large multicenter approach, GMA was part of the follow-up protocol of the Zika Rio de Janeiro cohort,^2^ which included infants prenatally exposed to acute maternal illness with rash and/or ZIKV infection. In addition, GMA was used to assess a select sample of infants with microcephaly after congenital ZIKV infection and a large group of age- and sex-matched neurotypical control infants for comparison. Specifically, we aimed (1) to describe global general movement patterns at 3 to 5 months’ postterm age (ie, fidgety movements) together with the age-specific concurrent motor repertoire (ie, detailed GMA) and (2) to relate the integrity of neural functions (through global and detailed GMA) to the time of prenatal exposure to maternal illness, preterm birth, and positive vs negative results on RT-PCR assays (for ZIKV infection), as well as relating to the head circumference *z* score in the case of microcephaly (ie, microcephaly severity) and to outcomes at age 12 months in the case of ZIKV-positive infants (ie, how many infants prenatally exposed to ZIKV were neurotypical developers and how many had adverse findings by age 1 year).

## Methods

The study was approved by institutional review boards at the Medical University of Graz (Graz, Austria), the SARAH network of rehabilitation hospitals (Belo Horizonte, Brazil), Fundação Oswaldo Cruz (Fiocruz) (Rio de Janeiro, Brazil), the University of São Paulo (São Paulo, Brazil), the University of Chicago (Chicago, Illinois), and the University of California (Los Angeles). Written informed consent for recording, storage, and scientific use of audiovisual data was obtained for all infants.

### Study Design

This was an observational cohort study composed of 2 groups of children. The study followed the Strengthening the Reporting of Observational Studies in Epidemiology (STROBE) reporting guideline. The first group included children exposed in utero to a maternal exanthematous illness during the ZIKV epidemic in Brazil; the second group included infants without any perinatal ZIKV exposure from the Graz University Audiovisual Research Database for the Interdisciplinary Analysis of Neurodevelopment (GUARDIAN).

### Setting

The study was conducted at the SARAH network of rehabilitation hospitals in Belo Horizonte and at Fiocruz in Rio de Janeiro (both in Brazil) and at the Medical University of Graz in Graz, Austria. Infant recruitment for ZIKV-exposed children was from February 1, 2016, and April 30, 2017. Infant follow-up for Bayley Scales of Infant and Toddler Development, Third Edition^12^ (Bayley-III) testing was performed until June 2018. Infants without ZIKV exposure were selected from the GUARDIAN database.

### Participants

A total of 444 infants were enrolled between February 1, 2016, and April 30, 2017, including 111 children prenatally exposed to an acute maternal illness with rash during the ZIKV epidemic and 333 neurotypical control children (57.7% male and mean [SD] age, 14 [2] weeks for both groups). Seventy-eight infants born to mothers with an acute illness with rash during pregnancy originated from the Rio de Janeiro Zika cohort^2^ (Fiocruz, Rio de Janeiro, Brazil). Two infants had microcephaly at birth; prenatal and perinatal data are published elsewhere.^2^ An additional 33 infants with microcephaly due to congenital Zika syndrome followed up at the SARAH network of rehabilitation hospitals in Belo Horizonte, Brazil, were included. Microcephaly was defined as previously described.^2^ All 35 infants with microcephaly were diagnosed as having disproportionate microcephaly and had clinical and imaging findings consistent with congenital Zika syndrome. Fifty-eight of 78 mothers (74.4%) in Rio de Janeiro tested positive for ZIKV by RT-PCR of blood, urine, or both at the time of rash. The remaining 20 infants were born to mothers with an acute exanthematous illness during pregnancy who tested negative by RT-PCR for ZIKV infection at the time of rash (Figure).

**Figure.**
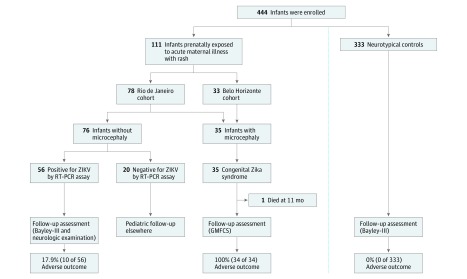
Prospective Cohort of Infants Prenatally Exposed to Acute Maternal Illness With Rash and Neurotypical Controls Adverse outcomes included neurologic deficits and/or abnormal Bayley Scales of Infant and Toddler Development, Third Edition (Bayley-III) results as defined in the text. Positive and negative for Zika virus by reverse transcriptase–polymerase chain reaction assay plus infants referred for congenital Zika syndrome. GMFCS indicates Gross Motor Function Classification System.

### Variables

For infant categorization, infants were enrolled in the exposure category if mothers had an acute illness with rash during pregnancy at the time of the ZIKV outbreak in Brazil. All 111 infants in the exposure category were matched for sex, gestational age at birth, and age at GMA to 3 infants without perinatal ZIKV exposure (n = 333) and with normal Bayley-III scores (Figure). Infants unexposed to ZIKV were randomly selected from the GUARDIAN database, which comprises more than 2000 standardized GMA data sets collected worldwide. Because general movements are endogenously generated motor patterns not influenced by culture or race/ethnicity,^9^ these variables were disregarded as matching criteria.

### General Movement Assessment

Details about GMA^8,9^ are provided in the eAppendix in the Supplement. At 9 to 20 weeks’ postterm age, infants were videotaped for 4 to 5 minutes of active wakefulness lying in supine position and without manipulation. All children whose mothers tested positive for ZIKV without microcephaly (n = 56) were reassessed at age 12 months. We chose Bayley-III^12^ for developmental testing because it is validated cross-culturally for Brazil.^13^ A score below 70 (ie, <2 SD) in the cognitive, language, or motor domain indicates severe developmental delay.^12^ We considered an adverse outcome as (1) a Bayley-III score less than 2 SD in at least 1 developmental domain, (2) a moderate developmental delay (<1 SD) in at least 2 domains (Bayley-III score <85), and/or (3) atypical neurologic findings. Infants of ZIKV-negative mothers (n = 20) did not have Bayley-III performed because they were referred elsewhere for pediatric follow-up once ZIKV infection was excluded. Children with microcephaly (n = 35) could not complete testing due to inability to follow directions. All control infants (n = 333) scored within normal range for all developmental domains (Figure).

### Data Sources and Measurement

The GMA and Bayley-III assessments for infants with exposure to maternal illness during pregnancy were conducted prospectively by study personnel in Brazil (F.U., C.Y.P.A., R.H.H., and F.F.G.), with the results recorded in case report forms. Infants without ZIKV exposure were selected from the GUARDIAN database in Graz. The same assessments were performed in both groups of children; study personnel in Brazil were trained by Graz investigators (C.E. and P.B.M.) to perform required study assessments previously obtained for database children.

### Bias

Study personnel performing GMA and interpreting the results (C.E., F.U., C.Y.P.A., C.P., R.H.H., F.F.G., and P.B.M.) were masked as to which infants had a confirmed diagnosis of antenatal ZIKV exposure vs maternal rash during pregnancy. Study personnel could not be masked to microcephaly; however, this was taken into consideration. All GMA videotapes were analyzed by at least 2 certified GMA experts (including C.I. and P.B.M.), with interscorer agreements of Cohen or Fleiss κ statistics ranging from 0.88 to 0.96 and intraclass correlation coefficients exceeding 0.90. The GMA experts were masked to the perinatal history, RT-PCR results, and final outcomes of participants. Staff members conducting developmental testing of the ZIKV cohort (C.E., F.U., and M.E.M.) were aware that toddlers were born to ZIKV-infected mothers, but had no information about GMA results. Children with microcephaly did not undergo developmental testing because they all had spastic cerebral palsy Gross Motor Function Classification System level IV, which renders them untestable. Masked assessment was not possible in children with microcephaly.

### Study Size

A total of 111 infants were enrolled in the maternal illness exposure cohort; this was the number of infants who came to clinic visits at the participating sites during the age window period necessary for GMA evaluations and whose parents or guardians provided written informed consent. All exposed infants were matched to 3 infants in the database without perinatal ZIKV exposure and with normal Bayley-III scores (n = 333), for a total sample size of 444 infants.

### Quantitative Variables

Quantitative variables were assessed. These included the Motor Optimality Score (MOS) and number of movement and postural patterns, apart from the core data, such as age, birthweight, and head circumference. The GMA data and Bayley-III scores were analyzed as categorical variables.

### Statistical Analysis

Using Pearson χ^2^ test or Fisher exact test (2 sided), we compared categorical data (clinical history and GMA) of infants who were prenatally exposed to acute maternal illness with rash (infants of mothers who were ZIKV RT-PCR positive or ZIKV RT-PCR negative and infants with microcephaly after congenital ZIKV infection) vs age-matched controls. To determine whether 2 independent samples (eg, ZIKV positive vs ZIKV negative, infants with congenital ZIKV vs controls, or normal vs adverse outcome) had the same distribution of their continuous variables (eg, MOS^9^), we applied the Mann-Whitney test. Two-sided *P* ≤ .05 was considered statistically significant. The sample Pearson product moment correlation coefficient *r* was applied to calculate the association between head circumference *z* scores (negative values) and continuous variables obtained through the detailed GMA (MOS and the number of movement or postural patterns). Sensitivity was calculated as [*a* / (*a* + *b*)] and specificity as [*d* / (*c* + *d*)], where *a* indicates abnormal or absent fidgety movements and adverse outcome (excluding microcephaly), *b* indicates normal fidgety movements and adverse outcome (excluding microcephaly), *c* indicates abnormal or absent fidgety movements and normal outcome, and *d* indicates normal fidgety movements and normal outcome.

## Results

While all 333 control infants had normal fidgety movements, none of the 35 infants with microcephaly developed this age-specific motor pattern. Among 76 infants in Rio de Janeiro without microcephaly, 64 (84.2%) had normal fidgety movements, 3 (3.9%) had abnormal exaggerated fidgety movements, and 9 (11.8%) did not show fidgety movements (Table 1). The rate of occurrence of aberrant fidgety movements (ie, abnormal or absent) was 15.8% (12 of 76) in infants prenatally exposed to acute maternal illness with rash but without microcephaly (9 infants of ZIKV-positive mothers and 3 infants of ZIKV-negative mothers) (Table 2) and 100% (35 of 35) in infants with ZIKV-induced microcephaly.

**Table 1.** Clinical Characteristics and Motor Behavior at 3 to 5 Months’ Postterm Age

Variable	Infants Without Microcephaly (n = 76)^a^	Infants With Microcephaly (n = 35)^b^	Neurotypical Controls (n = 333)	*P* Value^c^
Male, No. (%)	45 (59.2)	19 (54.3)	192 (57.7)	NA^d^
Preterm birth, No. (%)				
<34 wk gestation	2 (2.6)	0	6 (1.8)	NA^d^
34-36 + 6 wk gestation	8 (10.5)	5 (14.3)	39 (11.7)	
Weeks of gestation at the time of infection, wk, No. (%)				
≤13	22 (28.9)	29 (82.9)	NA	<.001^c^^,^^e^
14-28	42 (55.3)	6 (17.1)	NA	
≥29	12 (15.8)	0	NA	
Age at GMA, wk, No. (%)				
9-12	26 (34.2)	17 (48.6)	129 (38.7)	NA^d^
13-16	44 (57.9)	14 (40.0)	174 (52.3)	
17-20	6 (7.9)	4 (11.4)	30 (9.0)	
Fidgety movements, No. (%)^f^				
Normal	64 (84.2)	0	333 (100)	NA
Abnormal exaggerated	3 (3.9)	0	0	
Absent	9 (11.8)	35 (100)	0	
Motor Optimality Score				
Median (IQR) [range]	23 (19-26) [7-28]	5 (5-6) [5-9]	26 (24-28) [21-28]	<.001^g^
Optimal range of 25-28, No. (%)	22 (28.9)	0	231 (69.4)	<.001^e^
Reduced ≤24, No. (%)	54 (71.1)	35 (100)	102 (30.6)	
Repertoire, No. (%)				
Age adequate	35 (46.1)	1 (2.9)	246 (73.9)	<.001^e^
Not age adequate	41 (53.9)	34 (97.1)	87 (26.1)	
Movement patterns, apart from fidgety movements, median (IQR)				
Normal	3 (2-5)	0 (0-1)	5 (4-7)	<.001^g^
Abnormal	1 (0-2)	4 (3-6)	0 (0-1)	<.001^g^
Postural patterns, median (IQR)				
Normal	3 (2-3)	1 (1-1)	3 (3-4)	<.001^g^
Abnormal	2 (1-3)	6 (5-8)	1 (0-2)	<.001^g^
Movement character, No. (%)^h^				
Smooth and fluent	12 (15.8)	0	149 (44.7)	<.001^e^
Stiff	22 (28.9)	33 (94.3)	59 (17.7)	<.001^e^
Monotonous	38 (50.0)	34 (97.1)	89 (26.7)	<.001^e^
Cramped-synchronized^i^	0	19 (54.3)	0	NA

Abbreviations: GMA, general movement assessment; IQR, interquartile range; NA, not applicable.

^a^ Refers to 76 infants without microcephaly from the Rio de Janeiro cohort who were prenatally exposed to acute maternal illness with rash and whose mothers were referred to Fundação Oswaldo Cruz (Fiocruz) in Rio de Janeiro, Brazil. Prenatal and perinatal data are reported elsewhere.^2^

^b^ Refers to 33 infants from the Belo Horizonte cohort plus 2 infants with microcephaly from the Rio de Janeiro cohort.

^c^ The statistics compare infants without microcephaly vs infants with microcephaly.

^d^ Matching criteria.

^e^ Pearson χ^2^ test or Fisher exact test.

^f^ Normal fidgety movements are small movements of moderate speed with variable acceleration of the neck, trunk, and limbs in all directions; abnormal fidgety movements have a greater amplitude, speed, and jerkiness. Whenever fidgety movements are missing altogether from 9 to 20 weeks’ postterm age, this abnormality is called *absent fidgety movements*.^9^

^g^ Mann-Whitney test.

^h^ Because only the most frequently occurring movement characters are listed here, the percentages do not sum to 100%.

^i^ A cramped-synchronized movement characteristic denotes stiff movements; limb and trunk muscles contract almost simultaneously and then relax almost simultaneously.^9^

**Table 2.** Motor Behavior at 3 to 5 Months’ Postterm Age in the Rio de Janeiro Cohort (Excluding 2 Infants With Microcephaly) by Maternal Positive vs Negative Real-time RT-PCR Assays

Variable	Positive for ZIKV by RT-PCR Assay (n = 56)	Negative for ZIKV by RT-PCR Assay (n = 20)	*P* Value
Weeks of gestation at the time of infection, wk, No. (%)			
≤13	20 (35.7)	2 (10.0)	.03^a^
14-28	26 (46.4)	16 (80.0)	
≥29	10 (17.9)	2 (10.0)	
Fidgety movements, No. (%)			
Normal	47 (83.9)	17 (85.0)	.93^a^
Abnormal exaggerated	2 (3.6)	1 (5.0)	
Absent	7 (12.5)	2 (10.0)	
Motor Optimality Score			
Median (IQR) [range]	23 (18-26) [7-28]	23 (21-26) [7-27]	.57^b^
Optimal range of 25-28, No. (%)	16 (28.6)	6 (30.0)	.90^a^
Reduced ≤24, No. (%)	40 (71.4)	14 (70.0)	

Abbreviations: IQR, interquartile range; RT-PCR, reverse transcriptase–polymerase chain reaction; ZIKV, Zika virus.

^a^ Pearson χ^2^ test or Fisher exact test.

^b^ Mann-Whitney test.

Infants prenatally exposed to acute maternal illness who did not develop microcephaly (Rio de Janeiro cohort in the Figure) scored significantly lower on the MOS and all 5 subcategories compared with neurotypical controls. Infants with microcephaly (Belo Horizonte cohort and 2 infants with microcephaly from the Rio de Janeiro cohort) scored in the lowest range of the MOS and its 5 subscales (Table 1 and Table 3).

**Table 3.** Abnormal Postural Patterns

Variable	No. (%)			*P* Value^c^
	Infants Without Microcephaly (n = 76)^a^	Infants With Microcephaly (n = 35)^b^	Neurotypical Controls (n = 333)	
Head not kept in midline	22 (28.9)	30 (85.7)	64 (19.2)	.10
Asymmetric body posture	49 (64.5)	32 (91.4)	109 (32.7)	<.001
Persistent asymmetric tonic neck posture	0	9 (25.7)	0	NA
Hyperextension of neck and/or trunk	0	26 (74.3)	1 (0.3)	NA
Long-lasting extension of arms	12 (15.8)	6 (17.1)	38 (11.4)	.29
Long-lasting extension of legs	9 (11.8)	32 (91.4)	26 (7.8)	.26
External rotation and abduction of hips	20 (26.3)	1 (2.9)	11 (3.3)	<.001
Lack of variable finger movements	26 (34.2)	35 (100)	77 (23.1)	.08
Finger spreading	7 (9.2)	18 (51.4)	1 (0.3)	<.001
Spreading of toes	0	18 (51.4)	0	NA

Abbreviation: NA, not applicable.

^a^ Refers to 76 infants without microcephaly from the Rio de Janeiro cohort who were prenatally exposed to acute maternal illness with rash and whose mothers were referred to Fundação Oswaldo Cruz (Fiocruz) in Rio de Janeiro, Brazil. Prenatal and perinatal data are reported elsewhere.^2^

^b^ Refers to 33 infants from the Belo Horizonte cohort plus 2 infants with microcephaly from the Rio de Janeiro cohort.

^c^ Infants without microcephaly vs neurotypical controls (Pearson χ^2^ test or Fisher exact test).

Maternal illness occurred in the first trimester of pregnancy (1-12 weeks) in 82.9% (29 of 35) of infants diagnosed as having microcephaly compared with 28.9% (22 of 76) without microcephaly (*P* < .001) (Table 1). Aberrant fidgety movements in children without microcephaly exposed to maternal illness were more frequent after first-trimester exposure (58.3% [7 of 12]) (*P* = .04). The median MOS for infants exposed during the first trimester ranged from 21 (interquartile range [IQR], 11-26) to 24 (IQR, 20-26) when maternal illness occurred during the third trimester (*P* = .04). Five of 10 ZIKV-exposed infants with adverse outcomes at age 12 months were born to mothers with an acute illness and rash during the first trimester of pregnancy (cases 2, 5, 6, 7, and 9 in Table 4). Four had no fidgety movements; their MOS was lower (range, 7-17). The ZIKV-positive mothers (n = 56) had acute infection with rash more often in the first trimester of pregnancy than the ZIKV-negative mothers (n = 20) (*P* = .03) (Table 2). However, the infants’ neuromotor repertoire at 3 to 5 months was similar in infants of ZIKV-positive and ZIKV-negative mothers (Table 2).

**Table 4.** Brain Imaging, Fidgety Movements, and Motor Optimality Score, as Well as Outcome at Age 12 Months in Infants Exposed to ZIKV In Utero (Rio de Janeiro Cohort, Excluding 2 Infants With Microcephaly)

Case No.	Gestational Age at Birth, wk	Weeks of Gestation at the Time of ZIKV Infection, wk	Brain Imaging	Fidgety Movements	Motor Optimality Score	Outcome at Age 12 mo
1	35	27	IVH grade 1, small focus of periventricular cerebritis left, basal ganglia vasculopathy	Absent	10	Bayley-III <2 SD (language), hyperreflexia and hypertonia right > left
2	39	12	Normal	Absent	7	Bayley-III <2 SD (motor)
3	37	18	Normal	Absent	14	Bayley-III <1 SD (motor), dystonia, neurodevelopmental delay by neurologic examination
4	31	30	Normal	Absent	8	Bayley-III not available, neurologic and behavioral deficits
5	39	10	Brain atrophy, calcifications	Absent	8	Bayley-III not available, secondary microcephaly, neurologically impaired, seizures
6	36	11	Hydrocephaly	Absent	12	Bayley-III <1 SD (language), neurologically impaired
7	38	10	Normal	Absent	11	Bayley-III <1 SD (cognition and language)
8	39	38	IVH grade 1	Normal	28	Bayley-III <2 SD (language)
9	39	6	Normal	Normal	17	Bayley-III <2 SD (cognitive, language)
10	32	22	Periventricular calcifications, dolichocephaly	Normal	24	Bayley-III not available, macular hypoplasia, neurodevelopmental delay, hypertonia of lower limbs
11	38	29	Normal	Abnormal	14	Bayley-III <1 SD (language), neurologically normal
12^a^	37	12	Normal	Abnormal	11	Bayley-III normal, neurologically normal
13-56	NA	NA	Normal (29 of 44), abnormal (10 of 44), not done (5 of 44)	Normal (44 of 44)	17-28	Bayley-III normal (29 of 44), Bayley-III <1 SD in a single domain (10 of 44), Bayley-III not available (5 of 44), neurologically normal (44 of 44)

Abbreviations: Bayley-III, Bayley Scales of Infant and Toddler Development, Third Edition; IVH, intraventricular hemorrhage; NA, not applicable; ZIKV, Zika virus.

^a^ Fetal development was compromised by oligohydramnios.

There was no difference in gestational age at birth between infants exposed to acute maternal illness with or without microcephaly (Table 1). Neither the presence of aberrant fidgety movements nor the MOS was associated with preterm birth. However, both infants without microcephaly in the Rio de Janeiro cohort born at less than 34 weeks’ gestation (Table 1) had adverse outcomes at age 12 months (cases 4 and 10 in Table 4).

Four infants were diagnosed as having moderate microcephaly (*z* score <−2); the remaining 31 infants had severe microcephaly (*z* score <−3). The MOS was not associated with head circumference *z* scores (Pearson *r* = −0.048, *P* = .79). However, the more negative the *z* score was (ie, the smaller the head circumference), the more abnormal were the movement patterns (Pearson *r* = 0.370, *P* = .03). One patient developed secondary microcephaly (normocephalic at birth but with subsequent postnatal microcephaly) with absent fidgety movements; the MOS was 8 (case 5 in Table 4).

One infant with microcephaly due to congenital ZIKV infection died at 11 months. All 34 children with congenital microcephaly had bilateral spastic cerebral palsy with Gross Motor Function Classification System^14^ level V. They were unable to maintain antigravity head and trunk postures in prone and sitting positions and required caregiver assistance to roll. Twenty-seven of 34 children (79.4%) had epilepsy.

The neurodevelopmental outcomes of 56 children prenatally exposed to ZIKV without congenital microcephaly were abnormal for 10 children (cases 1-10 in Table 4); 82.1% (46 of 56) of ZIKV-exposed infants without congenital microcephaly were healthy at age 12 months. Forty-four of 46 infants (95.7%) whose development was considered normal at age 12 months (Bayley-III scores within normal range or <1 SD in a single domain with normal neurologic findings) had normal fidgety movements (*P* < .001) and were correctly identified by GMA at 3 months. Two children with abnormal exaggerated fidgety movements were considered normal at age 12 months (cases 11 and 12 in Table 4). The negative predictive value was 94% (95% CI, 85%-97%). Seven of 10 ZIKV-exposed children without microcephaly with adverse neurodevelopmental outcomes were identified by GMA. Of 10 ZIKV-exposed children without microcephaly with an adverse outcome at age 12 months, 7 were identified by absent fidgety movements (cases 1-7 in Table 4). The remaining 3 had normal fidgety movements (cases 8-10 in Table 4). The GMA positive predictive value was 78% (95% CI, 46%-94%). Sensitivity was 70% (95% CI, 35%-93%), and specificity was 96% (95% CI, 85%-99%). Accuracy was 91% (95% CI, 80%-97%). The MOS also differentiated between normal and adverse outcomes: children with normal outcomes whose mothers were ZIKV positive had a median MOS of 23 (IQR, 21-26), while children with an adverse outcome had a median MOS of 12 (IQR, 8-19) (*P* = .001). The median MOS in children with microcephaly was 5 (IQR, 5-6).

## Discussion

With the exception of a recent case report,^15^ our study is the first to perform a detailed functional analysis of the developing nervous system in infants with prenatal ZIKV exposure. We aimed to describe early motor functions and to search for potential markers of the neurodevelopmental outcomes at age 12 months after ZIKV exposure. We observed that infants aged 3 to 5 months prenatally exposed to ZIKV who did not develop congenital microcephaly moved less optimally compared with neurotypical controls. The number of normal age-specific movement patterns was reduced, and the quality of movements was altered (eAppendix in the Supplement). However, a similar observation was made in 20 infants whose prenatal exposure to acute maternal illness with rash could not be attributed to ZIKV and was possibly due to prenatal chikungunya exposure instead. The results of some studies suggest that chikungunya virus may compromise the developing nervous system in a similar but more subtle way.^16^

The most predictive movement patterns identified at 3 to 5 months’ postterm age are fidgety general movements. Infants with normal, continual fidgety movements are likely to develop neurotypically, whereas the lack of fidgety movements is a reliable marker for later neurologic deficits, most extensively associated with cerebral palsy.^8,10,11,17,18,19^ Because all 35 children with congenital microcephaly in the present study were diagnosed as having spastic cerebral palsy (Gross Motor Function Classification System level V) at around age 1 year, the lack of fidgety movements in this subgroup reflects the large body of evidence relating the absence of fidgety movements to development of cerebral palsy. Microscopic neuropathological changes described in infants with congenital microcephaly include necrosis-targeting neurons and degenerative changes of glia and neuronal cells, white matter loss, microcalcifications, and microglial aggregates,^20^ which translate into a lack of fidgety movements. Furthermore, we observed herein an association between head circumference and neurofunctional representation: the smaller the head circumference was, the more abnormal were the movement patterns noted. Apart from the absence of fidgety movements, infants with microcephaly had few normal movement patterns.

There were 9 infants among 76 in the nonmicrocephalic cohort (11.8%) who did not develop fidgety movements. Seven of them had confirmed prenatal exposure to ZIKV and an adverse outcome at age 12 months (cases 1-7 in Table 4). In 4 cases, the absence of fidgety movements identified infants at high risk of developing adverse outcomes, even with normal brain imaging results.

It is well known that maternal infections, particularly early in pregnancy, are associated with congenital microcephaly or other central nervous system abnormalities.^20,21^ Our study contributes to the discussion on the timing of maternal ZIKV infection because absent fidgety movements occurred more often when infants were exposed to maternal infection in the first trimester. Infants with a lack of fidgety movements attracted our attention because of their long-lasting wiggling-oscillating arm and/or leg movements, sometimes even wiggling neck movements, and occurring repetitive and/or long-lasting tongue protrusion.

To date, few studies have demonstrated that neurologic development of infants with congenital ZIKV infection can be abnormal in children without microcephaly.^22,23^ Cardoso and colleagues^23^ described neurologic signs in 19 infants born to mothers infected with ZIKV who did not develop microcephaly; 13 (68.4%) were initially seen with hypertonia. We observed stiff movements in one-third of the Rio de Janeiro cohort, including most infants with microcephaly. A stiff and/or monotonous movement characteristic also occurred in neurotypical controls, although less frequently. Ten-year outcomes of preterm infants demonstrated that children with monotonous, jerky, and/or stiff movement at 3 to 5 months have reduced cognitive functioning at school age.^24^ Eighty-six percent of infants (96 of 111) in our cohort were born at term, so it is unknown if a less optimal movement characteristic would be associated with school performance years later.

Apart from 333 neurotypical controls with normal fidgety movements at 3 to 5 months and a Bayley-III score exceeding 85 at their follow-up assessment (100% specificity), normal fidgety movements in the Rio de Janeiro cohort were associated with normal development at age 12 months, with a specificity of 96%. Conversely, the absence of fidgety movements was associated with an adverse outcome at age 12 months, with a sensitivity of 70%. Whereas the specificity coincides with that of previous studies^8,10,11^ irrespective of the outcome assessment age, the sensitivity in the present study is lower than the summary estimate (98%) for outcomes at 2 to 3 years^10,11^ but is in line with other studies reporting outcome assessments at age 12 months. With sensitivity ranging from 60% to 80%, the absence of fidgety movements was similar to that in our study and was related to lower scores in various developmental tests in a convenience high-risk sample in the United States,^25^ among infants after neonatal surgery in Australia,^26^ and in very low-birth-weight infants in China^27^ and India,^28^ as well as to any pathological findings in the 12-month neurologic examination.^19,29^ We acknowledge that an outcome at such a young age may be preliminary; some disorders might be diagnosed later in childhood, and milder forms of dysfunction may subsequently resolve. The predictive power of abnormal fidgety movements is known to be low,^8,9^ although a considerable number of infants with abnormal fidgety movements were described among individuals prenatally exposed to maternal opiate abuse and/or HIV who ultimately developed various neurodevelopmental and psychiatric impairments.^30^

Considering that ZIKV is strongly neurotropic, targeting not only neural progenitor cells but also neurons and other brain cells,^20^ our proportion of infants with normal fidgety movements at 3 to 5 months and normal neurodevelopment at age 12 months (78.6% [44 of 56]) was reassuring. We confirmed that GMA provides an opportunity to identify children with normal development. The high specificity of GMA is useful because it may help limit unnecessary referral of infants to heavily burdened rehabilitation services, especially in low- and middle-income countries. Implementing GMA may also facilitate prompt referral to such services, which can optimize development, prevent secondary complications, and enhance family well-being. In this context, we emphasize that GMA is easy to apply and is a cost-effective method, especially for low- and middle-income countries.^9,28^

### Limitations

Given the study complexity, there were limitations related to data availability. For our comprehensive assessment of endogenously generated motor behavior during the first months of life, we assessed 44.8% (56 of 125) of the original Zika Rio de Janeiro cohort.^2^ The main reason for the reduced sample size is that infants were followed up at different sites throughout Rio de Janeiro, which reduced patient availability for GMA assessments. In addition, GMA evaluations were performed at strict intervals, and often infants were either too young or too old within the time frame for this assessment. Another study limitation is that many mothers with negative ZIKV RT-PCR results opted not to continue long-term developmental follow-up of their infants. For this reason, Bayley-III assessments were not available for infants of ZIKV-negative mothers.

## Conclusions

Our study provides insight into the spectrum of both neurologic sequelae and normal outcomes observed from age 3 to 12 months in children prenatally exposed to maternal ZIKV infection. Most ZIKV-exposed children without microcephaly (82.1% [46 of 56]) were classified as healthy at age 12 months. Because the true rate of transmission of ZIKV from mother to child is unknown, the association of GMA with sequelae due to ZIKV could actually be superior to the results reported herein if the population of infants evaluated was composed solely of infected infants rather than exposed. Further investigation of long-term sequelae in ZIKV-exposed infants, such as late presenting developmental disorders, is required. Our findings demonstrate that children with adverse outcomes can be reliably identified by GMA, which is a valid complementary assessment tool. We recommend its incorporation for routine assessment of infants prenatally exposed to acute maternal illnesses. General movement assessment will help identify infants who may benefit from targeted intervention programs.

## Notes

Supplement. **eAppendix.** Supplemental Appendix
