# Supplementary material for: Association of Infants Exposed to Prenatal Zika Virus Infection With Their Clinical, Neurologic, and Developmental Status Evaluated via the General Movement Assessment Tool
Source: JAMA Netw Open. 2019 Jan 18;2(1):e187235. doi: 10.1001/jamanetworkopen.2018.7235 (PMC6431234; doi:10.1001/jamanetworkopen.2018.7235)
Supplement: Supplement. — eAppendix. Supplemental Appendix [file jamanetwopen-2-e187235-s001.pdf]

Supplementary Online Content

Einspieler C, Utsch F, Brasil P, et al; GM Zika Working Group. Association of infants exposed to prenatal Zika virus infection with their clinical, neurologic, and developmental status evaluated via the general movement assessment tool. *JAMA Netw Open*. 2019;2(1):e187235.
doi:10.1001/jamanetworkopen.2018.7235

eAppendix. Supplemental Appendix

This supplementary material has been provided by the authors to give readers additional information about their work.

© 2019 Einspieler C et al. *JAMA Network Open*.

eAppendix. Supplemental Appendix

The General Movement Assessment

Since its introduction some 20 years ago,[1] GMA has been applied to study and clinically assess infants with acquired brain injury, or after neonatal surgery,[2] in the evaluation of newborns with intrauterine growth restriction, and infants who were prenatally exposed to adverse maternal health conditions or drug abuse.[e.g.,1,2, 3,4, ,5] The GMA provides 98% sensitivity for the prediction of cerebral palsy (95% CI 74-100%),[3,4,6] and also identifies infants at high risk for (a) minor neurological dysfunction, (b) certain genetic and neurodevelopmental disorders with a diagnosis in or beyond toddlerhood (e.g., Rett syndrome, autism spectrum disorder), or (c) learning difficulties at school age). Summary estimates of its specificity are 91% (95% CI 83-93%).[3]

GMA can be applied from birth onwards,[6] but its high predictive power primarily lies in the assessment of the fidgety movements, which is the age-specific general movement pattern of three- to five-month-old infants. Numerous studies and meta-analyses have confirmed the initial published findings,[1] i.e., that infants develop normally if their fidgety movements are normal, even if their clinical history may indicate a disposition to later neurological deficits. Conversely, almost all infants who never develop fidgety movements are at high risk for an adverse outcome.[3,4,7,8] Thus, GMA is one of the tools of choice when it comes to predicting neurological outcomes.

The GMA is based on visual Gestalt perception of videoed age-specific normal and abnormal movement patterns with interrater reliabilities ranging from 0.88 to 0.92 (Kappa values).[4],[6]During the postterm age of three to five months, GMs appear as fidgety movements, which are small movements of the neck, trunk, and limbs in all directions and of variable acceleration[1,6] indicating a normal neurological development (LR– = 0.04; 95% CI: 0.005 to 0.27). Abnormal fidgety movements with exaggerated amplitude, speed and jerkiness may point to neurological deficits, but their predictive power is low (LR+ = 5.1; 95% CI: 1.5 to 17).[1] It is the absence of fidgety movements that is strongly related to the development of severe neurological deficits (LR+ = <51), most intensively studied as a predictor of cerebral palsy.[814, 9]

In addition to fidgety movements, we assessed in detail other movement patterns and postures specific for the age of three to five months. The detailed GMA, also called the "Assessment of Motor Repertoire at Three to Five Months of Age,"[6, p. 26] comprises the following five sub-categories: (i) temporal organization of fidgety movements, (ii) age-adequacy of motor repertoire, (iii) quality of movement patterns other than fidgety movements, (iv) posture, and (v) overall quality of the motor repertoire. It reveals a motor optimality score (MOS) with a maximum of 28 (best possible performance) and a minimum of five. An MOS from 25 to 28 is considered to be optimal; scores ≤24 are reduced.[6] The inter-observer reliability is high with intra-class correlation coefficients ranging from 0.80 to 0.94. [1]

Results (additional)

The motor optimality score, MOS

Neither country of origin nor ethnicity (defined by the parents of the child and the investigators) affected the MOS of the control infants: infants born in South America (median=26; IQR=24–26) did not differ from infants born in North America or Europe (median=26; IQR=24–28; p=0.505); Caucasian infants (median=26; IQR=24–28) did not differ from non-Caucasians (median=26; IQR=24–26; p=0.685). Overall, the median MOS of the control group was with 26 within the optimal range (Table 1). These results emphasised once again[6] that endogenously generated movement patterns are independent of ethnicity, environmental settings and care-giving procedures.

The number of normal movement patterns (co-occurring with fidgety movements) ranged from 2 to 14 (median 5) in control infants, from 0 to 10 (median 3) in the Rio de Janeiro cohort, and from 0 to 2 in infants with microcephaly (median 0; Table 1). The most frequently occurring normal movement patterns in control infants were fidgety movements (100%), foot-to-foot contact (62%), visual scanning (57%), smiling (38%), hand-to-mouth contact (36%), wiggling-oscillating arm movements (32%), hand-to-hand contact (32%), legs lift (32%), and kicking (30%). Each of these movement patterns occurred significantly less often in infants prenatally exposed to acute maternal infection with rash who did not develop microcephaly (p values ranging from 0.037 to 0.001). The few age-specific movement patterns observed in infants with microcephaly were smiling (14%), and foot-to-foot contact (6%) or side-to-side movements of the head (6%).

A small proportion of control infants exhibited a few abnormal movement patterns. Among those were long lasting and/or repetitive tongue protrusion (8%) and abnormal foot-to-foot contact (i.e. both feet frequently striking each

© 2019 Einspieler C et al. *JAMA Network Open*.

other on the tibial side without any plantar contact; 5%). Abnormal tongue protrusion also occurred in 14 infants of the Rio de Janeiro cohort (18%) and in nine infants with microcephaly (26%). Apart from tongue protrusion, the most frequently occurring abnormal movement patterns in the Rio de Janeiro cohort were long-lasting wiggling-oscillating movements of arms, neck and/or legs (33%) and abnormal eye movements, such as strabismus or slow pursuit (9%). Abnormal eye movements were also seen in 24 infants with microcephaly (69%). In addition, they showed circular arm movements (46%), long lasting stiff head anteflexion (40%), en bloc trunk rotations (31%), long-lasting kicking (29%), and/or monotonous side-to-side movements of the head (26%). Ten infants (29%) with microcephaly were almost hypokinetic in the lower limbs, which also occurred in two infants (3%) of the Rio de Janeiro cohort who did not develop microcephaly.

Seventy-five percent of all control infants had four normal postural patterns such as head in midline, symmetric body posture, absence of asymmetric tonic neck posture, and variable finger postures, whereas 75% of the infants from the Rio de Janeiro cohort had only three normal postural patterns (Tables 1 and 3 of the main manuscript). Infants with microcephaly had only one normal postural pattern.

The majority of infants with microcephaly (86%) could not maintain the head in midline, whereas no difference regarding this item was observed between the Rio de Janeiro cohort and controls (29 vs. 19%; Table 3 of the main manuscript). Long-lasting extensions of arms and/or legs were also almost equally often observed in the Rio de Janeiro cohort and the controls. Long-lasting extension of the legs, however, occurred in 91% of infants with microcephaly ($p<0.001$). A persistent asymmetric tonic neck posture, hyperextension of the neck and/or trunk, and spreading of toes merely occurred in infants with microcephaly (Table 3 of main manuscript). None of the infants with microcephaly had variable finger postures; 13 infants (37%) were constantly fisting and the remaining 22 infants (63%) had rarely occurring monotonous finger movements. External rotation and abduction of the hip hardly occurred in infants with microcephaly but was present in 26% of the Rio de Janeiro cohort. A cramped-synchronised movement character (i.e. stiff limb and trunk muscles contract almost simultaneously and then relax almost simultaneously)[6] occurred only in infants with microcephaly (19/35; 53%). A monotonous movement character occurred in all three groups; it was present in 27% of the control infants, in every second infant of the Rio de Janeiro cohort, and in almost all infants with microcephaly (Table 1 of main manuscript). Tremulous movements occurred in only one infant of the Rio de Janeiro cohort and in 13 infants with microcephaly (37%). In any case the monotonous, jerky and/or stiff movement character observed in the control group did not correlate with the Bayley-III scores at 12 to 30 months as their scores were in the normal range. The described irritability and tremulousness [e.g.,10,11] in ZIKV-exposed non-microcephalic infants was not confirmed in our study. The explanation might be that previous observations referred mainly to neonates whereas we included three to five-month-olds.

Only two of 35 infants showed normal foot-to-foot contact and normal side-to-side movements of the head; another five infants socially smiled. Apart from these few normal patterns, infants with microcephaly showed abnormal eye movements (strabismus, nystagmus), long lasting stiff head anteflexion, *en bloc* trunk rotations, circular arm movements, monotonous and long lasting (mainly unilateral) kicking or no leg movements at all; if the legs were not moving they were kept in full extension. A cramped-synchronised movement character was observed in every second infant with microcephaly. Such rigid movements with almost simultaneous contraction of at least two limbs and the trunk followed by an almost simultaneous relaxation is known to be highly predictive for severe spastic cerebral palsy,[1] especially if it persists beyond two months after term. Finally, none of the infants with microcephaly exhibited variable finger postures. This is in line with previous observations about the association between variable finger postures and a normal cognitive development.[12]

<u>References</u>

1. Prechtl HF, Einspieler C, Cioni G, Bos AF, Ferrari F, Sontheimer D. An early marker for neurological deficits after perinatal brain lesions. *Lancet.* 1997;349(9062):1361-1363.
2. Crowle C, Galea C, Walker K, et al. Prediction of neurodevelopment at one year of age using the general movement assessment in the neonatal surgical population. *Early Hum Dev.* 2018;118:42-47.
3. Bosanquet M, Copeland L, Ware R, Boyd R. A systematic review of tests to predict cerebral palsy in young children. *Dev Med Child Neurol.* 2013;55(5):418-426.
4. Novak I, Morgan C, Adde L, et al. Early, accurate diagnosis and early intervention in cerebral palsy. Advances in diagnosis and treatment. *JAMA Pediatr.* 2017;171(9):897-907.

© 2019 Einspieler C et al. *JAMA Network Open*.

5. Palchik AB, Einspieler C, Evstafeyeva IV, Talisa VB, Marschik PB. Intra-uterine exposure to maternal opiate abuse and HIV: the impact on the developing nervous system. *Early Hum Dev.* 2013;89(4):229-235.
6. Einspieler C, Prechtl HFR, Bos AF, Ferrari F, Cioni G. Prechtl's method on the qualitative assessment of general movements in preterm, term and young infants. *Clin Dev Med.* 2004;167:1-104.
7. Burger M, Louw QA. The predictive validity of general movements – a systematic review. *Eur J Paediatr Neurol.* 2009;13(5):408-420.
8. Darsaklis V, Snider LM, Majnemer A, Mazer B. Predictive validity of Prechtl's method on the qualitative assessment of general movement: a systematic review of the evidence. *Dev Med Child Neurol.* 2011;53(10):896-906.
9. Morgan C, Crowle C, Goyen TA, et al. Sensitivity and specificity of general movement assessment for diagnostic accuracy of detecting cerebral palsy in an Australian context. *J Paediatr Child Health.* 2016;52(1):54-59.
10. van der Linden V, Pessoa A, Dobyns W, et al. Description of 13 infants born during October 2015-January 2016 with congenital Zika virus infection without microcephaly at birth – Brazil. *MMWR Morb Mortal Wkly Rep.* 2016;65(47):1343-1348.
11. Cardoso TF Jr, dos Santos RS, Corrēa RM, et al. Congenital Zika infection: neurology can occur without microcephaly. *Arch Dis Child.* 2018; doi:10.1136/archdischild-2018-314782
12. Einspieler C, Bos AF, Libertus ME, Marschik PB. The general movements assessment helps us to identify preterm infants at risk for cognitive dysfunction. *Front Psychol* 2016;7:406. doi:10.3389/fpsyg.2016.00406.

© 2019 Einspieler C et al. *JAMA Network Open*.
